# The continuance intention to vaccinate against COVID-19: An empirical study from Malaysia

**DOI:** 10.1371/journal.pone.0301383

**Published:** 2024-04-30

**Authors:** Li-Ann Hwang, Santha Vaithilingam, Jason Wei Jian Ng, Mahendhiran Nair, Pervaiz Ahmed, Kamarul Imran Musa

**Affiliations:** 1 Department of Business Analytics, Sunway Business School, Sunway University, Selangor, Malaysia; 2 Sunway Institute for Global Strategy and Competitiveness, Sunway University, Selangor, Malaysia; 3 Department of Applied Statistics, School of Mathematical Sciences, Sunway University, Selangor, Malaysia; 4 Department of Community Medicine, School of Medical Sciences, Universiti Sains Malaysia, Kelantan, Malaysia; St John’s University, UNITED STATES

## Abstract

**Background:**

Vaccination has been one of the most effective preventive strategies to contain the COVID-19 pandemic. However, as the COVID-19 vaccines’ effect wanes off after some time and given their reduced level of protection against mutation strains of the virus, the calls for boosters and second boosters signal the need for continuous vaccination for the foreseeable future. As Malaysia transitions into the endemic phase, the nation’s ability to co-exist with the virus in the endemic phase will hinge on people’s continuance intention to be vaccinated against the virus. Adapting the expectations confirmation model (ECM) to the public health context and in a developing country, this study integrates the ECM with the health belief model (HBM) and the theory of reasoned action (TRA) to examine the inter-relationships of the predictors of people’s continuance intention to vaccinate against COVID-19.

**Methodology:**

Data were collected using self-administered questionnaires from 1,914 respondents aged 18 and above by a marketing consulting firm via its online panel. The partial least squares structural equation modeling (PLS-SEM) technique was used to analyze the data.

**Results:**

Out of the 1,914 respondents, 55.9% reported having a continuance intention to vaccinate against COVID-19, similar to other developing countries. The multivariate analysis revealed that perceived usefulness and satisfaction significantly influenced individuals’ continuance intention to vaccinate against COVID-19. Additionally, attitude was found to play a key role in influencing behavioral change among individuals towards their perceptions of continuously getting vaccinated against COVID-19.

**Conclusions:**

By integrating three theoretical frameworks (i.e., HBM, TRA and ECM), this study showed that behavioral characteristics could provide insights towards continuance vaccination intention. Hence, policymakers and key stakeholders can develop effective public health strategies or interventions to encourage vaccine booster uptake by targeting behavioral factors such as perceived usefulness, attitude, satisfaction, and subjective norms.

## Introduction

Since the World Health Organization’s (WHO) declaration of the coronavirus disease (COVID-19) outbreak as a global pandemic in early 2020, the COVID-19 pandemic has been a protracted battle for numerous countries. The successive waves of coronavirus outbreaks experienced by numerous countries reveal the ongoing and evolving health (physical and mental) and economic challenges governments confront to contain the pandemic [1–3].

In the initial response to the pandemic, many countries opted for strict movement controls by implementing nationwide lockdowns and border closures to mitigate the chain of transmissions. However, with the subsequent vaccine development [4] and increased immunization of people populations, the ensuing reduction in new cases and deaths [5] has enabled countries to ease COVID-19 restrictions, such as relaxing their border and movement restrictions and doing away with mask mandates. Hence, although non-pharmaceutical interventions such as physical distancing, wearing masks, and targeted or full lockdowns are standard public health tools used to mitigate the pandemic, vaccination against COVID-19 is arguably the most effective preventive strategy to contain the pandemic [6–8].

Nevertheless, there has been mounting evidence that the COVID-19 vaccines’ effect wanes off after some time, whereby the antibody levels decline progressively after the primary round of vaccinations [9–13]. In addition, there is also evidence that the available vaccines cannot offer the same level of protection against mutation strains of the virus [14, 15]. For example, the Pfizer-BioNTech vaccine was only 88% effective against symptomatic disease due to the B.1.617.2 variant two weeks after the second dose, but 93% effective against the B.1.1.7 variant. Meanwhile, the AstraZeneca vaccine was 60% effective against symptomatic disease due to the B.1.617.2 variant after two doses compared to 66% effectiveness against the B.1.1.7 variant [16]. The estimated vaccine effectiveness was lower for the omicron variant for infections, hospitalisations, and mortality at baseline compared with that of other variants, but subsequent reductions occurred at a similar rate across variants [17].

In response to this development, a booster dose has been advocated after the primary vaccination dosage [18, 19]. Furthermore, a second booster for vulnerable persons has recently been recommended by the WHO’s Strategic Advisory Group of Experts on Immunization [20]. Therefore, as the virus continues to evolve and mutate, continuous vaccination is becoming more likely in the near future. This is necessary to avoid a resurgence of infections that may lead to the return of drastic movement control measures that have been shown to cause mental health problems [21–23] and negative economic impacts [24].

In line with the WHO recommendations, Malaysia also began offering booster doses to its population. However, as of May 17, 2023, only 50% of the total population has received their booster dose [25]. Furthermore, only 6.1% of the elderly aged 60 and above have taken their second booster dose [25]. These statistics significantly contrast with the high initial COVID-19 vaccination intention rates [26, 27] that have resulted in 84.4% of the total population completing their primary round of COVID-19 vaccination [25]. Therefore, while the population demonstrated a strong willingness to receive their initial COVID-19 vaccinations, their inclination to receive booster doses appears considerably lower. Given the nation’s transition into the endemic phase since April 1, 2022 [28], the nation’s ability to co-exist with the virus in the endemic phase will hinge on people’s continuance intention to be vaccinated against the virus [29].

This study aims to address a significant knowledge gap by examining and identifying the predictors of the Malaysian adult population’s continuance intention to be vaccinated against COVID-19. It is important to note that, while previous Malaysian research by Wong et al. [6] has explored this related topic, our study differentiates itself by encompassing a broader scope. Rather than solely focusing on the initial booster dose, we extend our inquiry to encompass a commitment to sustained vaccination efforts essential for effectively controlling the pandemic. Furthermore, previous studies that have probed into people’s willingness to receive booster doses have primarily assessed factors such as attitude and satisfaction. Nevertheless, these studies have not empirically examined the relationship between these factors and the intention to receive subsequent booster doses [30–32]. In contrast, our study takes a distinctive and integrated approach by analyzing people’s continuance intention to be vaccinated, drawing on well-established health behavior models such as the Health Belief Model (HBM) and the Theory of Reasoned Action (TRA) [33, 34]. In addition, we incorporate the Expectation Confirmation Model (ECM), which was initially derived from the consumer behavior literature to conceptualize a model for continued information systems usage intention [35].

The ECM posits that consumers tend to establish initial expectations for a product before making a purchase [35]. Subsequently, they form perceptions about the product’s performance and assess it against their initial expectations. Consumers who are satisfied with the product are then more likely to develop repurchase intentions towards the product. Similarly, the ECM can elucidate continuance intention regarding COVID-19 vaccination. It suggests that individuals’ initial expectations formed during their primary vaccine doses will allow them to shape their perception towards vaccination. As individuals experience the benefits and outcomes of their initial vaccination, they may adjust their expectations, perceptions, and satisfaction levels, which can, in turn, influence their intention to continue receiving COVID-19 vaccines.

As continuous vaccination is key to avoiding the resurgence of infections, it is important to identify and develop a thorough understanding of its key predictors. This aids in ensuring the viability of the nation’s endemicity, especially when there are signs that high levels of population immunity are associated with reduced severity of COVID-19, similar to that of seasonal influenza [36]. As such, studying the continuance intention to get vaccinated, especially in a developing country, becomes necessary, as context-specific effects play a central role in comprehending phenomena and helping researchers in explaining observed findings [37]. The findings from this study would provide insights to policymakers and health authorities about the relevant programs and initiatives to encourage vaccination not only for COVID-19 vaccines but other immunization efforts as well.

## Literature review

The COVID-19 pandemic has brought the issue of vaccination to the forefront, with numerous studies exploring various aspects of vaccination intention and behavior. Historically, much of the research in this area has been focused on understanding the *initial* intention to vaccinate against COVID-19 [26, 38]. However, the dynamics of COVID-19 vaccination extend beyond the initial decision, warranting an investigation into continuous vaccination behaviors.

Despite the critical importance of ongoing vaccination efforts, empirical studies addressing this specific aspect have been relatively scarce. A systematic review conducted by Galanis et al. [39] identified only 14 studies related to the acceptance of the initial COVID-19 booster dose, reflecting a limited focus on continued vaccination behaviors. In a more recent systematic review, Ayyalasomayajula et al. [40] included 42 articles relevant to vaccine booster hesitancy, indicating a growing interest in the topic.

Some of the few studies that have ventured into the realm of continuous vaccination intention include those by Kunno et al. [30] for Thailand, Abuhammad et al. [31] and Al-Qerem et al. [41] for Jordan, Rzymski et al. [32] for Poland, and Galanis et al. [39] for Greece. These studies offer valuable insights into regional variations and concerns about continuous vaccination behavior, shedding light on the broader global context.

In the Malaysian context, Wong et al. [6] investigated pandemic fatigue, alongside standard demographic and attitude factors, among other variables, expanding the scope of factors considered in understanding vaccination behavior. Chang et al. [42], on the other hand, primarily examined demographic factors and attitudes towards COVID-19 booster vaccination among the Malaysian population.

Notably, most of these studies lacked a well-defined theoretical framework to justify their selection of predictors. They relied on descriptive and diagnostic analytics as their primary analysis tool, which may limit the depth of insight into the factors influencing continuous vaccination behavior. Moreover, these studies often centred around demographic characteristics, attitudes, and satisfaction without explicitly modeling the relationships between these factors and the intention for continuous vaccination.

Furthermore, a common limitation in prior research is snowball sampling, which may introduce selection bias into the study population. In contrast, this study leveraged the partnership of a reputable market research company to collect data through a nationwide survey via stratified sampling, enhancing the representativeness of the sample and the robustness of the findings.

## Conceptual framework

**Fig 1:**
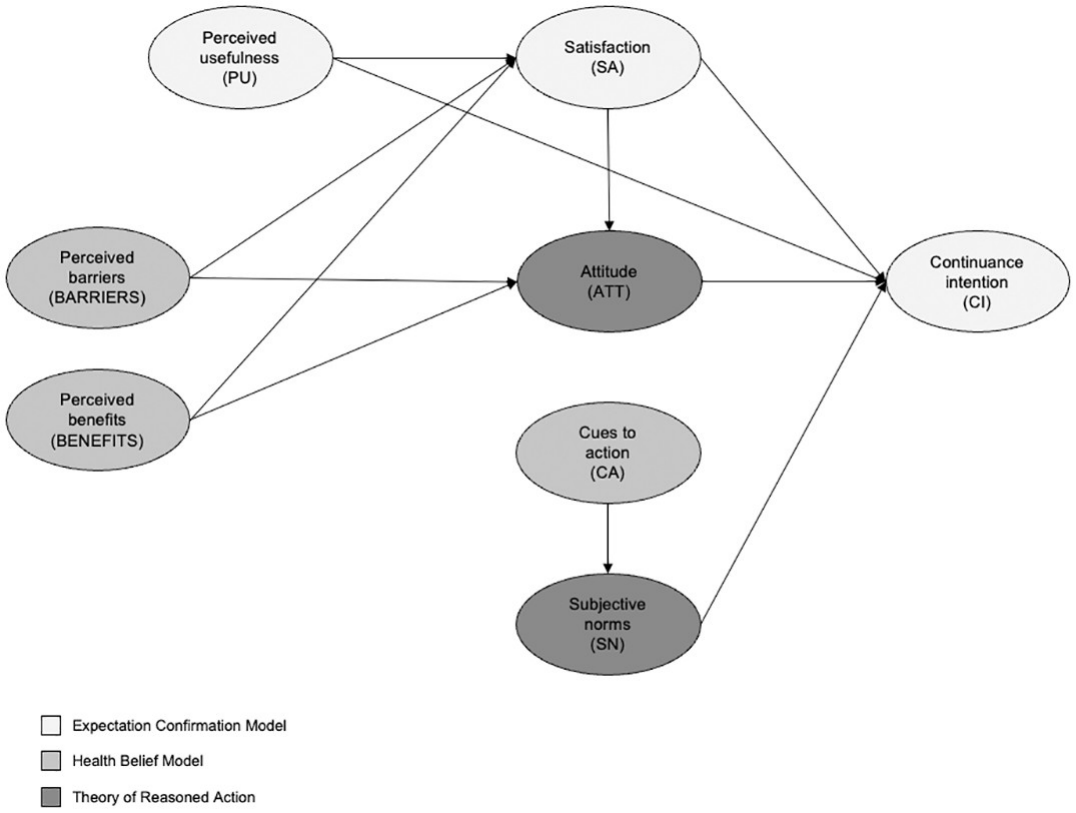
Conceptual framework.

This study integrates the HBM, TRA and ECM to examine individuals’ continuance intention to vaccinate against COVID-19 (as shown in Fig 1). The HBM is a widely used theoretical framework to predict health behaviors, including COVID-19 vaccination intention [27, 43, 44]. The HBM comprises the following constructs: perceived susceptibility, perceived severity, perceived benefits, perceived barriers and cues to action. However, this study will only consider three of these constructs: perceived barriers, perceived benefits, and cues to action. Perceived susceptibility and severity were not included as individuals may not be as concerned about the severity of or their susceptibility to the virus compared to the early stages of the pandemic when there was limited knowledge about COVID-19. With the pandemic approaching its fourth year, most individuals would have received their primary vaccination dosage or been exposed to the virus, contributing to higher immunity against the virus. As a result, individuals are not as concerned about the health risks arising from infection [45]. However, without continued vaccination and immunization against the virus, the immunity of prior vaccinations will decline over time [45]. This study highlights the need to consider other factors that may have gained prominence over time.

To complement the HBM, a value-expectancy theory, we include the TRA in our conceptual framework as it includes key social cognitive variables crucial in predicting behavior [46]. In particular, the TRA postulates that an individual’s continuance intention to get vaccinated is dependent on their attitude toward vaccinations and subjective norms.

In addition to the HBM and TRA, this study borrows from the information systems literature by including the ECM. The ECM was adapted from consumer behavior studies and applied to information systems research to examine individuals’ continuance intention to use technology [35]. Recognizing that the long-term feasibility of a system and its subsequent success depends on its continued use rather than initial use [35], the development of the ECM was a progression from earlier technology adoption models that largely examined the initial adoption of technology. In the same spirit, this study’s inclusion of the ECM to investigate individuals’ continuance intention to vaccinate against COVID-19 is a progression from earlier studies that examined individuals’ initial COVID-19 vaccination intention. Based on the ECM, we hypothesize that individuals’ satisfaction with and their perceived usefulness of COVID-19 vaccines predict their continuance intention to vaccinate against COVID-19. As individuals in our study’s sample would have already completed their primary round of vaccination, we included the construct measuring satisfaction as their evaluation of the initial vaccination experience will enable them to form perceptions that could influence their subsequent vaccination behavior. Additionally, perceived usefulness is expected to bring about utilitarian benefits of vaccination such as improved health and well-being which in turn will prompt individuals to engage in continuous vaccination behavior.

## Methodology

### Study design

A nationwide cross-sectional study was conducted in Malaysia from January 17 to January 26, 2022. A locally based international marketing consulting firm was engaged to collect data from Malaysian adults aged 18 years and above using self-administered questionnaires via its online panel. The marketing consulting firm’s online panel consists of 32,000 panelists throughout the country whereby the gender, age group and regional distribution are aligned with the Malaysian population census distribution. A representative stratified sample of respondents was selected and contacted via email.

The sample size was determined using the inverse square root method proposed by Kock and Hadaya [47] and can be expressed using the following formula:

$$\hat{N}>{\left(\frac{2.486}{{\left|\beta \right|}_{min}}\right)}^{2}$$

where *N* is the sample size and |*β*|_*min*_ is the absolute value of the statistically significant path coefficient with the minimum magnitude.

Assuming a 5% significance level, the minimum sample size estimated using the formula above is 1,298. This method of calculating the required minimum sample size is fairly precise for normal and non-normal data [47].

### Measures

The questionnaire was designed using validated instruments from prior studies. To establish the validity of all measures, we employed previously validated items [48], making slight adjustments as needed to align with the specific context of this study. In this study, our key variable of interest is the participants’ continuance intention to receive the COVID-19 vaccine, defined as an individual’s intention to vaccinate against COVID-19 continuously.

Based on the HBM, we incorporated three constructs to measure participants’ perceived barriers and benefits of vaccinating against COVID-19 and cues to action. Perceived benefits is defined as the positive outcomes of getting vaccinated [33]. Perceived barriers, conceptualized as access and clinical barriers, refer to individuals’ assessment of the influences that impede or discourage vaccination [33]. Cues to action refer to the strategies to activate readiness which leads to the execution of the behavior [33]. Items measuring these constructs were obtained from several sources [34, 49, 50].

Drawing upon the TRA, we included items measuring attitude and subjective norms from Chu & Liu [49] and Yang [34]. This study defines attitude as one’s evaluative affect on getting vaccinated for COVID-19 continuously [51]. Subjective norms refer to one’s perception about whether most people who are important to them think they should or should not continuously get the COVID-19 vaccine [51].

In addition to the HBM and TRA, we adapted items from Zhu et al. [7] that measure the ECM constructs given by satisfaction, perceived usefulness, and continuance intention. Satisfaction is defined as the positive experience from completing the primary round of COVID-19 vaccination, while perceived usefulness refers to the utilitarian value of vaccinating against COVID-19 which includes indicators measuring health, well-being, and convenience [7, 35]. The full list of items with their respective sources are provided in S1 Appendix. All items were measured on a 5-point Likert scale.

We also obtained data about participants’ sociodemographic characteristics, such as age, income, education level, employment status, marital status, and whether they had previously been infected with COVID-19.

The questionnaire was developed in English and translated into Malay and Chinese by professional translators. The Malay and Chinese questionnaires were back-translated to ascertain that the original and translated questionnaires were similar in content and meaning.

### Data analysis method

The partial least squares structural equation modelling (PLS-SEM) technique is extensively utilized in exploratory studies and is recognized as a second-generation technique capable of addressing measurement error, modelling complex structures, and accommodating non-normal data distribution [52]. SmartPLS 4 [53] was utilized to analyze the interrelationships between the constructs.

Statistical tests such as the Shapiro-Wilk and Kolmogorov-Smirnov are commonly employed to evaluate the normality of the data. However, skewness and kurtosis values should be examined in addition to normality [52, 54]. According to Hair et al. [52], skewness and kurtosis values that lie between -2 and + 2 are generally considered acceptable. Based on the evaluation of skewness and kurtosis, the results show that the indicators are skewed. Despite the indicators being skewed, PLS-SEM is still robust in the presence of non-normality.

### Data analysis

Data analyses were conducted using two statistical softwares. R version 4.2.1 was used for descriptive analysis where p-values of less than 0.05 were considered statistically significant. Multivariate analysis was performed using the PLS-SEM technique given its advantage of the model obtaining high predictive accuracy and concurrently based on causal explanations [55, 56]. PLS-SEM is a regression-based model and has been gaining traction as a method of choice for exploring complex inter-relationships between observed and latent constructs [52].

### Ethics statement

We obtained ethics approval from the Monash University Human Research Ethics Committee (Project ID: 28249). Online written consent was obtained from the participants before the questionnaire was administered. The research team ensured that any personal identifiers of the participants were de-identified.

## Results

### Sociodemographic characteristics

A total of 1,914 respondents took part in the nationwide survey, with the central region having the majority of respondents and the east coast region the smallest (36% vs 9%). Table 1 shows the overall sociodemographic characteristics of the respondents. The ratio of females to males is almost one (0.96), 75% of respondents were aged 50 years or younger, and nearly half (47%) received tertiary education. Over half were Malay respondents (53%) and married (55%), and 14% had been infected with COVID-19 before the survey was conducted.

**Table 1:** Characteristics of participants.

Sociodemographic characteristic	Overall, N = 1,914	Female, n = 937 (49%)	Male, n = 977 (51%)
**Age Group, n(%)**			
18–30	565 (30%)	340 (36%)	225 (23%)
31–40	449 (23%)	239 (26%)	210 (21%)
41–50	428 (22%)	185 (20%)	243 (25%)
51–60	311 (16%)	123 (13%)	188 (19%)
Above 60	161 (8.4%)	50 (5.3%)	111 (11%)
**Income, n(%)**			
Less than RM1,000	344 (18%)	209 (22%)	135 (14%)
RM1,000—RM3,999	718 (38%)	352 (38%)	366 (37%)
RM4,000—RM6,999	440 (23%)	210 (22%)	230 (24%)
RM7,000—RM9,999	262 (14%)	115 (12%)	147 (15%)
Above RM10,000	150 (7.8%)	51 (5.4%)	99 (10%)
**Education, n(%)**			
Secondary or lower	497 (26%)	224 (24%)	273 (28%)
Diploma	513 (27%)	271 (29%)	242 (25%)
Tertiary Education	904 (47%)	442 (47%)	462 (47%)
**Employment, n(%)**			
Disabled	5 (0.3%)	2 (0.2%)	3 (0.3%)
Domestic homemaker	81 (4.2%)	76 (8.1%)	5 (0.5%)
Employed full time	976 (51%)	459 (49%)	517 (53%)
Employed part time	119 (6.2%)	67 (7.2%)	52 (5.3%)
Retired	158 (8.3%)	67 (7.2%)	91 (9.3%)
Self-Employed	336 (18%)	127 (14%)	209 (21%)
Student	143 (7.5%)	90 (9.6%)	53 (5.4%)
Unemployed, looking for work	78 (4.1%)	38 (4.1%)	40 (4.1%)
Unemployed, not looking for work	18 (0.9%)	11 (1.2%)	7 (0.7%)
**Ethnicity, n(%)**			
Malay/Bumiputera	1,019 (53%)	501 (53%)	518 (53%)
Chinese	732 (38%)	356 (38%)	376 (38%)
Indian/Others	163 (8.5%)	80 (8.5%)	83 (8.5%)
**Marital Status, n(%)**			
Married	1,051 (55%)	463 (49%)	588 (60%)
Never married	759 (40%)	420 (45%)	339 (35%)
Divorced/Separated	83 (4.3%)	39 (4.2%)	44 (4.5%)
Widowed	21 (1.1%)	15 (1.6%)	6 (0.6%)
**Had COVID-19 before, n(%)**			
Yes	267 (14%)	105 (11%)	62 (17%)
No	1647 (86%)	832 (89%)	815 (83%)
**Region, n(%)**			
Central*	696 (36%)	343 (37%)	353 (36%)
East Coast*	172 (9.0%)	84 (9.0%)	88 (9.0%)
East Malaysia*	269 (14%)	144 (15%)	125 (13%)
North*	416 (22%)	175 (19%)	241 (25%)
South*	361 (19%)	191 (20%)	170 (17%)

*Central: Kuala Lumpur, Putrajaya, Selangor

*East Coast: Kelantan, Pahang, Terengganu

*East Malaysia: Sabah, Sarawak

*North: Kedah, Penang, Perak, Perlis

*South: Johor, Malacca, Negeri Sembilan

Table 2 reports the sociodemographic characteristics of respondents grouped according to their continuance intention to vaccinate against COVID-19: Unlikely, Neutral and Likely. Based on the reported Chi-squared test p-values, income, education, employment, ethnicity, and marital status are sociodemographic characteristics that influence respondents’ intention to be continually vaccinated against COVID-19.

**Table 2:** Sociodemographic characteristics of respondents according to continuance intention groups.

Sociodemographic characteristic	Unlikely, n = 225 (11.7%)	Neutral, n = 620 (32.4%)	Likely, n = 1,069 (55.9%)	p-value^1^
**Gender, n(%)**				0.8
Female	106 (47%)	303 (49%)	528 (49%)	
Male	119 (53%)	317 (51%)	541 (51%)	
**Age Group, n(%)**				0.6
18–30	57 (25%)	183 (30%)	325 (30%)	
31–40	53 (24%)	152 (25%)	244 (23%)	
41–50	55 (24%)	130 (21%)	243 (23%)	
51–60	36 (16%)	98 (16%)	177 (17%)	
Above 60	24 (11%)	57 (9.2%)	80 (7.5%)	
**Income, n(%)**				0.011
Less than RM1,000	43 (19%)	126 (20%)	175 (16%)	
RM1,000—RM3,999	93 (41%)	229 (37%)	396 (37%)	
RM4,000—RM6,999	50 (22%)	156 (25%)	234 (22%)	
RM7,000—RM9,999	25 (11%)	63 (10%)	174 (16%)	
Above RM10,000	14 (6.2%)	46 (7.4%)	90 (8.4%)	
**Education, n(%)**				0.004
Secondary or lower	70 (31%)	185 (30%)	242 (23%)	
Diploma	62 (28%)	158 (25%)	293 (27%)	
Tertiary Education	93 (41%)	277 (45%)	534 (50%)	
**Employment, n(%)**				<0.001
Disabled	3 (1.3%)	0 (0%)	2 (0.2%)	
Domestic homemaker	12 (5.3%)	36 (5.8%)	33 (3.1%)	
Employed full time	95 (42%)	315 (51%)	566 (53%)	
Employed part time	17 (7.6%)	35 (5.6%)	67 (6.3%)	
Retired	22 (9.8%)	44 (7.1%)	92 (8.6%)	
Self-Employed	56 (25%)	99 (16%)	181 (17%)	
Student	12 (5.3%)	48 (7.7%)	83 (7.8%)	
Unemployed, looking for work	6 (2.7%)	32 (5.2%)	40 (3.7%)	
Unemployed, not looking for work	2 (0.9%)	11 (1.8%)	5 (0.5%)	
**Ethnicity, n(%)**				<0.001
Malay/Bumiputera	146 (65%)	334 (54%)	539 (50%)	
Chinese	54 (24%)	250 (40%)	428 (40%)	
Indian/Others	25 (11%)	36 (5.8%)	102 (9.5%)	
**Marital Status, n(%)**				0.013
Married	148 (66%)	331 (53%)	572 (54%)	
Never married	62 (28%)	259 (42%)	438 (41%)	
Divorced/Separated	12 (5.3%)	24 (3.9%)	47 (4.4%)	
Widowed	3 (1.3%)	6 (1.0%)	12 (1.1%)	
**Had COVID-19 before, n(%)**				0.4
Yes	27 (12%)	81 (13%)	159 (15%)	
No	198 (88%)	539 (87%)	910 (85%)	
**Region, n(%)**				0.11
Central^2^	69 (31%)	228 (37%)	399 (37%)	
East Coast^3^	24 (11%)	61 (9.8%)	87 (8.1%)	
East Malaysia^4^	32 (14%)	96 (15%)	141 (13%)	
North^5^	58 (26%)	137 (22%)	221 (21%)	
South^6^	42 (19%)	98 (16%)	221 (21%)	

^1^Chi-squared test

^2^Kuala Lumpur, Putrajaya, Selangor

^3^Kelantan, Pahang, Terengganu

^4^Sabah, Sarawak

^5^Kedah, Penang, Perak, Perlis

^6^Johor, Malacca, Negeri Sembilan

### Continuance intention to vaccinate against COVID-19

Table 3 shows the means and standard deviations (SDs) for the nine HBM, TRA, and ECM constructs for respondents grouped according to their continuance intention to vaccinate against COVID-19: Unlikely, Neutral and Likely. ANOVA tests show strong and statistically different means for all variables. A positive correlation is observed between continuance intention to vaccinate and the following six variables: individual benefits, community benefits, attitude, subjective norms, satisfaction, and perceived usefulness. The Likely group observed a higher mean score for these variables. On the other hand, there is a negative association for clinical and access barriers with continuance intention, whereby a higher mean score is associated with the Unlikely group.

**Table 3:** Means and SDs of HBM, TRA, and ECM constructs for respective continuance intention groups.

Variable	Unlikely, n = 225^1^	Neutral, n = 620^1^	Likely, n = 1,069^1^	p-value^2^
Clinical barriers	3.68, (0.82)	3.34, (0.68)	3.03, (0.94)	<0.001
Access barriers	2.88, (0.99)	2.97, (0.82)	2.68, (1.03)	<0.001
Individual benefits	3.16, (0.94)	3.61, (0.68)	4.21, (0.69)	<0.001
Community benefits	3.47, (0.98)	3.85, (0.65)	4.39, (0.59)	<0.001
Cues to action	3.34, (0.83)	3.34, (0.75)	3.58, (0.81)	<0.001
Attitude	3.05, (1.09)	3.74, (0.79)	4.50, (0.60)	<0.001
Subjective norms	3.51, (1.02)	3.97, (0.74)	4.57, (0.56)	<0.001
Satisfaction	2.95, (1.06)	3.70, (0.71)	4.42, (0.58)	<0.001
Perceived usefulness	2.82, (0.99)	3.66, (0.67)	4.46, (0.54)	<0.001

^1^Mean, (SD);

^2^One-way ANOVA

Note: Refer to S3 Appendix for post-hoc tests identifying which categories differ from each other for each of the nine variables.

The difference in means between the Unlikely and Likely group of respondents for attitude, satisfaction and perceived usefulness is approximately 1.5, indicating that these constructs play a more significant role in influencing one’s continuance intention to vaccinate against COVID-19.

### Multivariate analysis of the predictors of COVID-19 continuance intention

This study adopted the two-step procedure introduced by Anderson and Gerbing [57] to analyze the measurement and structural models. The measurement model was evaluated to ensure reliability, convergent validity, and discriminant validity. Once this has been established, we assessed the structural model that displays the relationship between the constructs [52].

All constructs were measured using reflective indicators. Perceived barriers was conceptualized as a reflective-reflective higher-order construct (HOC). Including HOCs results in greater parsimony, allowing a better understanding of the model [58].

Table 4 shows the results from the evaluation of the measurement model. Internal consistency reliability is measured using Cronbach’s *α* and composite reliability (CR). The CR values are preferred over the Cronbach’s *α* as the Cronbach’s *α* assumes that the indicators are unweighted and hence may be less reliable [55]. Table 4 shows that the CR values on all constructs are above the 0.70 threshold, implying adequate internal consistency reliability [55]. To assess indicator reliability, we refer to the values of the standardized outer loadings. Most outer loadings have a value greater than the threshold value of 0.70. Indicator loadings between 0.40 and 0.70 were retained as they were important in measuring their respective constructs [52]. The AVE values in Table 4 are all above 0.50, suggesting that convergent validity has been achieved.

**Table 4:** Measurement model evaluation.

Construct	Indicator	Outer Loadings	Cronbach’s *α*	CR	AVE
ATT	ATT1	0.901	0.892	0.933	0.823
	ATT2	0.917			
	ATT3	0.903			
BARRIERS	ABR	0.814	0.609	0.830	0.710
	CBR	0.870			
BENEFIT	CBENF1	0.868	0.909	0.931	0.694
	CBENF2	0.890			
	CBENF3	0.726			
	IBENF1	0.767			
	IBENF2	0.962			
	IBENF3	0.759			
CA	CA1	0.574	0.777	0.841	0.519
	CA2	0.727			
	CA3	0.807			
	CA4	0.830			
	CA5	0.629			
CI	CI1	0.943	0.943	0.964	0.898
	CI2	0.957			
	CI3	0.943			
PU	PU1	0.906	0.871	0.921	0.795
	PU2	0.889			
	PU3	0.879			
SA	SA1	0.906	0.899	0.937	0.832
	SA2	0.918			
	SA3	0.913			
SN	SN1	0.893	0.941	0.955	0.810
	SN2	0.888			
	SN3	0.920			
	SN4	0.930			
	SN5	0.867			

Note: CR–Composite reliability; AVE–Average variance extracted; ATT–Attitude; BARRIERS–Perceived barriers; BENEFITS–Perceived benefits; CA–Cues to action; CI–Continuance intention; PU–Perceived usefulness; SA–Satisfaction; SN–Subjective norms.

Table 5 reports the measurement model evaluation of the higher-order construct, perceived barriers. Based on the values of the outer loadings, Cronbach’s *α*, CR, and AVE, we have adequately established indicator reliability, internal consistency reliability and convergent validity.

**Table 5:** Measurement model evaluation of higher- and lower-order constructs.

Higher-order construct (HOC)					
**HOC**	**LOC**	**Outer Loadings**	**Cronbach’s *α***	**CR**	**AVE**
BARRIERS	ABR	0.814	0.609	0.830	0.710
	CBR	0.870			
Lower-order constructs (LOCs)					
**LOC**	**Indicators**	**Outer Loadings**	**Cronbach’s *α***	**CR**	**AVE**
ABR	ABR1	0.868	0.858	0.903	0.701
	ABR2	0.885			
	ABR3	0.818			
	ABR4	0.774			
CBR	CBR1	0.732	0.789	0.864	0.613
	CBR2	0.771			
	CBR3	0.838			
	CBR4	0.788			

Note: ABR–Access barriers; BARRIERS–Perceived barriers; CBR–Clinical barriers.

To establish discriminant validity between the constructs, we evaluate the heterotrait-monotrait (HTMT) ratio of correlations. Table 6 presents the HTMT results. The correlation estimates are below 0.90, and the 95% bootstrap confidence intervals do not contain the value of 1, indicating discriminant validity between the constructs [59]. Discriminant validity is not considered between the higher-order construct, perceived barriers, and its respective lower-order constructs as the repeated indicators approach was used. Using the repeated indicators approach, the higher-order construct repeats the indicators of its associated lower-order constructs, and therefore it is expected that discriminant validity cannot be established [60].

**Table 6:** Discriminant validity.

	ABR	ATT	BARRIERS*	BENEFIT	CA	CBR	CI	PU	SA
ATT	**0.259** [0.212, 0.307]								
BARRIERS*	**-**	**0.374** [0.327, 0.420]							
BENEFIT	**0.151** [0.100, 0.206]	**0.621** [0.578, 0.663]	**0.200** [0.158, 0.254]						
CA	**0.072** [0.053, 0.114]	**0.165** [0.110, 0.217]	**0.094** [0.076, 0.134]	**0.361** [0.306, 0.415]					
CBR	**0.530** [0.482, 0.579]	**0.379** [0.331, 0.425]	**-**	**0.195** [0.159, 0.248]	**0.090** [0.062, 0.144]				
CI	**0.143** [0.092, 0.196]	**0.662** [0.624, 0.697]	**0.280** [0.228, 0.332]	**0.577** [0.533, 0.618]	**0.173** [0.120, 0.227]	**0.330** [0.278, 0.380]			
PU	**0.181** [0.129, 0.236]	**0.713** [0.673, 0.751]	**0.274** [0.226, 0.329]	**0.724** [0.673, 0.751]	**0.266** [0.684, 0.762]	**0.283** [0.226, 0.329]	**0.773** [0.673, 0.751]		
SA	**0.225** [0.173, 0.278]	**0.715** [0.677, 0.750]	**0.313** [0.262, 0.366]	**0.668** [0.625, 0.706]	**0.258** [0.202, 0.311]	**0.306** [0.259, 0.358]	**0.691** [0.653, 0.725]	**0.869** [0.838, 0.897]	
SN	**0.166** [0.118, 0.216]	**0.631** [0.582, 0.677]	**0.183** [0.141, 0.233]	**0.662** [0.617, 0.704]	**0.258** [0.203, 0.311]	**0.156** [0.120, 0.207]	**0.558** [0.514, 0.602]	**0.661** [0.617, 0.704]	**0.639** [0.593, 0.683]

Note:

1. The values in bold are the correlation estimates, whereas the values in the square brackets are the 95% confidence intervals.

2. ABR–Access barriers; ATT–Attitude; BARRIERS–Perceived barriers; BENEFITS–Perceived benefits; CA–Cues to action; CBR–Clinical barriers; CI–Continuance intention; PU–Perceived usefulness; SA–Satisfaction; SN–Subjective norms.

3. * Represent the higher-order construct of perceived barriers.

To uncover unobserved heterogeneity issues, we employed the finite mixture PLS (FIMIX-PLS) procedure. Generally, the FIMIX-PLS procedure allows researchers to reliably reveal the existence of heterogeneity using the information criterion [61]. Specifically, we focus on Akaike’s information criterion with factor 3 (AIC_3_) and consistent AIC (CAIC). The results show that AIC_3_ and CAIC produce different results in terms of segment sizes, which is an indication of the absence of unobserved heterogeneity issues (see S2 Appendix) [62].

**Fig 2:**
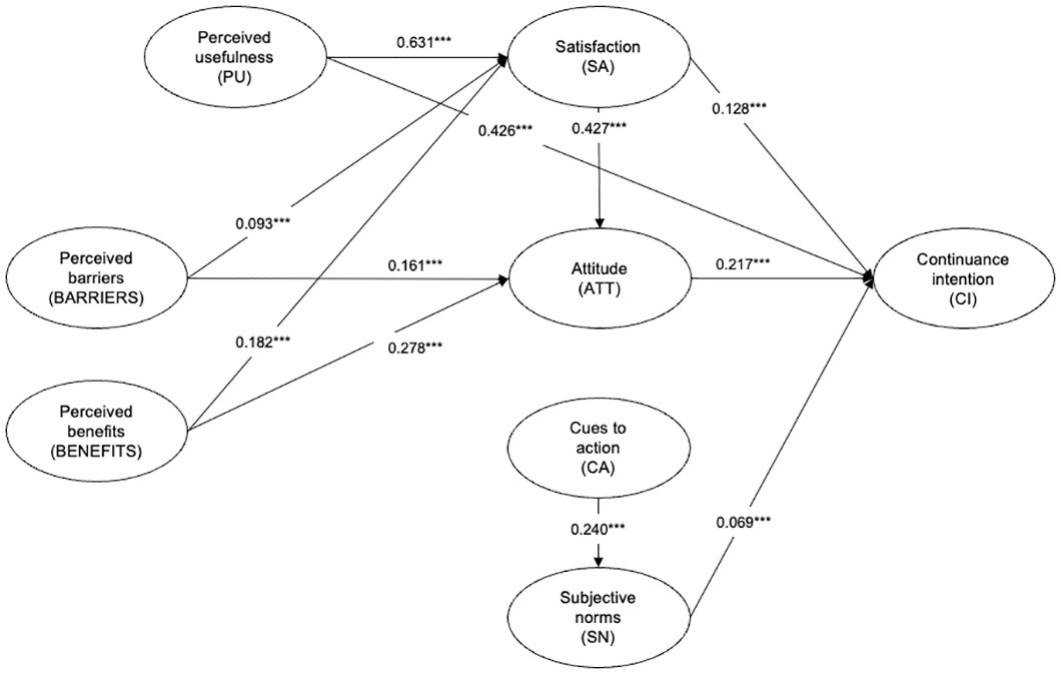
Conceptual framework with structural model results. Note: *** p<0.01.

Fig 2 illustrates the results of the structural model. The two HBM constructs used in this study, perceived barriers and perceived benefits, significantly influenced an individual’s satisfaction and attitude toward continually vaccinating for COVID-19. Both constructs from the TRA, attitude and subjective norms, influenced one’s continuance intention to get vaccinated. For the ECM constructs, satisfaction significantly influenced attitude and continuance intention, while perceived usefulness influenced satisfaction and continuance intention.

## Discussion

Many countries worldwide, including Malaysia, have transitioned to the endemic phase, gradually learning to co-exist with the virus. International borders have reopened and the physical distancing requirements and mask mandates, removed. Nevertheless, given that the protection provided by vaccines diminishes over time, accompanied by the unpredictable nature of the virus’ mutation, it is vital to understand individuals’ behavior to continuously get vaccinated against the virus.

Based on our sample results, 55.9% of Malaysian adults aged 18 and above have a continuance intention to vaccinate against COVID-19, including the booster dose. This result is similar to other developing countries such as Algeria (51.6%) [63] and Saudi Arabia (55.3%) [64]. However, the acceptance rates of the booster dose are higher in developed countries (e.g., Japan and Italy), ranging from 85% to 98% [65–67]. Therefore, behavioral insights derived from our study are a step forward to improving the nation’s intention to continually vaccinate against the virus, thereby strengthening its ability to co-exist with the virus in its endemic phase.

Drawing upon three theoretical models from the HBM, TRA and ECM, our multivariate analysis illuminated four insights into the behavioral factors influencing people’s continuance intention to vaccinate.

First, the direct path relationship between perceived usefulness and continuance intention to vaccinate appeared to be the strongest. In our study’s context, the indicators used to measure vaccination’s perceived usefulness relate to health, well-being and convenience. Therefore, if individuals perceive COVID-19 vaccines as having strong protection against the virus and that it improves their well-being through conveniences and privileges accorded to them due to vaccination (e.g., the ability to travel or dine in), they are more likely to maintain a continuance intention to vaccinate against COVID-19. Consequently, individuals’ strong perception of the utility of vaccination plays an integral role in influencing their continuance intention to be vaccinated. This finding is consistent with Zhu et al. [7], who found that the perceived usefulness of the vaccine is a prominent factor in influencing people’s emotions and subsequent behaviors.

Second, our results highlight the prominent role of attitude, an integral factor in spearheading behavioral change, that could influence individuals’ continuous intention to vaccinate against COVID-19. This result is consistent with prior literature that has identified attitude as a salient factor influencing vaccine booster acceptance across various countries such as Jordan, Poland, and the United States [31, 68, 69]. Even for the vaccine-hesitant, attitude has been shown to play a critical role in influencing these individuals’ intention to vaccinate against COVID-19 [70].

Given the significant impact attitude has on continuance intention, multiple studies have focused on sociodemographic factors as possible predictors of attitude. For example, Jairoun et al. [71] showed that demographic variables such as gender, education level, and employment status were associated with greater positive attitudes towards getting a vaccine booster. In contrast, this study conceptualized attitude as a combined function of the HBM and ECM. Accordingly, our results show that perceived barriers, perceived benefits, and satisfaction affect individuals’ attitudes towards their continuance intention to vaccinate against COVID-19. Specifically for the HBM constructs of perceived barriers and benefits, our findings are similar to Hu et al. [43]. The authors found that greater perceived benefits will lead to a greater willingness to get the vaccine booster. They attribute this observation to China’s extensive effort to promote booster shots to strengthen one’s immunity against the virus [43]. This finding is also similar in Jordan, where individuals were more likely to get vaccine boosters if they perceived them to be beneficial in protecting themselves and their community against COVID-19 [31].

Third, satisfaction has a significant influence on individuals’ continuance vaccination intention. In the information systems literature, individuals’ satisfaction with a product indicates the extent of fulfilment of their expectations, leading to continued use of the product [72]. Hence, adopting this line of reasoning in the COVID-19 context, individuals who have completed their primary round of vaccination are more likely to have a favorable view of continuous vaccination if they had a positive experience during the vaccination process. This indicates that satisfaction with prior vaccination experience could lead to more positive attitudes towards vaccination and, subsequently, greater continuance intention to receive COVID-19 vaccination. As countries transition to an endemic phase, individuals may be more concerned about the level of satisfaction than the severity or susceptibility to the virus compared to the early stages of the pandemic. Therefore, one way to target behavioral change to encourage continuous vaccination could be by enhancing individuals’ vaccination experience.

However, our finding on satisfaction contradicts the results from a study conducted in China by Zhu et al. [7], who found that satisfaction did not significantly impact continuance intention. The authors posited that vaccination behaviors are complex due to diverse individual perceptions. For example, the authors opined that individuals in their study may still be skeptical about the efficacy of vaccines in protecting themselves against the virus and thus do not find it necessary to get vaccinated. In contrast, our study focuses on individuals who have already received their primary vaccine doses and may display different perceptions regarding vaccines.

Fourth, although subjective norms was also significant in predicting continuance intention, its low path coefficient value indicates its diminished role when compared to perceived usefulness, satisfaction and attitude. This finding is a significant departure from prior research where subjective norms has been one of the most salient factors in predicting individuals’ initial intention to receive the COVID-19 vaccine [70, 73, 74]. Thus, our results allude to how the role of subjective norms transpires in importance when moving from initial vaccination to continuous vaccination. The opinion of important others towards vaccination behavior is influential during the initial stages. However, as individuals gain experience and realize the benefits of vaccination, this would allow them to make informed decisions about their subsequent vaccination behavior. Therefore, subjective norms may not be as crucial in predicting continuance intention.

The results have important public policy implications in enhancing the continuation of vaccination against COVID-19. The results suggest that policymakers must consider behavioral characteristics such as perceived usefulness, the attitude of people towards vaccination, satisfaction level, and subjective norms (i.e., the role of family, friends, and people who are important to the individual) in their COVID-19 vaccination strategies. In this context, community engagement and public awareness campaigns [e.g., Communication, Education and Public Awareness (CEPA)] targeted towards increasing continued vaccination must clearly highlight the benefits of ongoing vaccination. The benefits to individuals include keeping them healthy, ensuring they become immune to future infections and improving their overall quality of life. All of which will enhance their satisfaction level with the vaccination process.

Further, for the CEPA programs to be effective in a multiethnic country such as Malaysia, these programs must be conducted in languages spoken by the diverse population in the country and jointly undertaken with social influencers such as family, friends, and people of importance to the targeted subjects. These social influencers play a significant role in the decision-making process, and can increase vaccination rates and continued vaccinations in the population [75]. Among the key people of importance is community leaders (e.g., village heads and religious leaders) who play a key role in influencing people in their own communities to make important decisions. The role of community leaders was found to be more important for people from minority, rural, and vulnerable communities in making decisions on their vaccination status [76]. As such, targeted CEPA programs can be developed for social influencers in effectively ‘nudging’ their family, friends, and people in their communities to remain immune against emerging variants of the COVID-19 virus by continuously updating their vaccination status.

This study has several limitations. First, data collection was conducted via an online panel skewed towards the urban population. Therefore, the findings may limit generalizability to the overall population. Future research could consider obtaining a representative sample from the rural population for the findings to be generalizable to the overall population. Second, the current study employs a cross-sectional design where an individual’s perception of continuance intention was obtained from a single point in time. Considering this limitation, future studies could seek to undertake a longitudinal study as individual perceptions may change over time due to various factors. A longitudinal study would be able to provide worthwhile insights into individual behavior and external conditions that may affect one’s continuance intention to vaccinate. Despite the limitations, the results of this study were able to provide insights into individuals’ continuous vaccination intentions, which can be applicable to policymakers in formulating strategies and interventions not only for COVID-19 but for future pandemics as well.

## Conclusion

Although the COVID-19 pandemic will soon enter its fourth year, discussion on vaccine hesitancy is still ongoing, considering the waning effects of vaccines and the emergence of new variants. Continuous vaccination is, therefore, vital to avoid the resurgence of the virus, highlighting the need for vaccine boosters.

This study integrated three theoretical frameworks (i.e., HBM, TRA and ECM) to examine the factors influencing continuous vaccination intention among Malaysians that can provide valuable insights to shape public health strategies to ensure immunity against the virus. Our findings accentuate the importance of satisfaction and perceived usefulness in determining continuous vaccination intention. The findings also revealed the vital role of attitude as an agent of behavioral change among individuals for continuous vaccination. Based on these findings, policymakers and key stakeholders can formulate strategies and interventions to encourage vaccine booster uptake by improving satisfaction and changing individual attitudes by promoting the inherent benefits of vaccines and leveraging the influence of community leaders.

## Supporting information

S1 Appendix List of items. (DOCX)

S2 Appendix FIMIX-PLS results. (DOCX)

S3 Appendix Tukey’s test. (DOCX)

S1 Checklist STROBE statement—Checklist of items that should be included in reports of observational studies. (DOCX)
