# Supplementary material for: The continuance intention to vaccinate against COVID-19: An empirical study from Malaysia
Source: PLoS One. 2024 Apr 30;19(4):e0301383. doi: 10.1371/journal.pone.0301383 (PMC11060549; doi:10.1371/journal.pone.0301383)
Supplement: S1 Appendix — (DOCX) [file pone.0301383.s001.docx]

## S1 Appendix. List of items

| **Construct** | **Indicator** | **Statement of indicators** | **Source** |
| --- | --- | --- | --- |
| ATT |  | **Using the following adjective scales, please indicate how much you feel that getting vaccinated for Covid-19 is:** | Chu & Liu, 2021; Yang, 2015 |
|  | ATT1 | 1 "Unfavourable" to 5 "Favorable" |  |
|  | ATT2 | 1 "Bad" to 5 "Good" |  |
|  | ATT3 | 1 "Harmful" to 5 "Beneficial" |  |
| BARRIERS (ACCESS) | ABR1 | It is inconvenient to get the Covid-19 vaccine as I have no control over the time of vaccination appointment. | Coe et al., (2012); Yang (2015) |
|  | ABR2 | It is inconvenient to get the Covid-19 vaccine as I have no control over the venue for my vaccination. |  |
|  | ABR3 | The Covid-19 vaccine is not easily accessible to me |  |
|  | ABR4 | It is inconvenient to do a walk-in appointment for my booster vaccination. |  |
| BARRIERS (CLINICAL) | CBR1 | My body may react adversely to the Covid-19 vaccine | Yang, 2015; Chu & Liu, 2021; Coe et al., 2012 |
|  | CBR2 | I will get sick from the Covid-19 vaccine |  |
|  | CBR3 | I am concerned about whether Covid-19 vaccines are safe |  |
|  | CBR4 | I feel that not enough research has been done on Covid-19 vaccines |  |
| BENEFIT | CBENF1 | Having myself vaccinated against Covid-19 is beneficial for the health of others in my community | Chu & Liu (2021) |
|  | CBENF2 | Covid-19 vaccines protect the health of my community |  |
|  | CBENF3 | Covid-19 vaccines will enable free travel within and outside of the country |  |
|  | IBENF1 | Covid-19 vaccines will work in preventing the disease |  |
|  | IBENF2 | Covid-19 vaccines will be effective in preventing Covid-19 |  |
|  | IBENF3 | If I get the vaccines, I will be less likely to get Covid-19 |  |
| CA |  | **In the past 1 year, how much attention have you paid to news related to Covid-19 from:** | Yang (2015) |
|  | CA1 | Printed newspaper |  |
|  | CA2 | Television |  |
|  | CA3 | Social media such as Instagram, Facebook, TikTok, Twitter, YouTube, etc. |  |
|  | CA4 | Internet (e.g. news site portals; Google) |  |
|  | CA5 | Radio |  |
| CI | CI1 | I am likely to continue to get vaccinated against COVID-19 in the future, if needed. | Zhu et al. (2021) |
|  | CI2 | I intend to consistently get vaccinated against COVID-19 in the future, if needed. |  |
|  | CI3 | I intend to continue getting vaccinated against COVID-19 rather than discontinue the vaccine. |  |
| PU | PU1 | I think that the COVID-19 vaccine protects my health. | Zhu et al. (2021) |
|  | PU2 | Getting vaccinated against COVID-19 improves my well-being. |  |
|  | PU3 | Getting vaccinated against COVID-19 helps me to go about my daily activities conveniently. |  |
| SA | SA1 | Overall, I am satisfied with the COVID-19 vaccine. | Zhu et al. (2021) |
|  | SA2 | The COVID-19 vaccine I am currently getting meets my expectations. |  |
|  | SA3 | I am very pleased with my experience with getting vaccinated against COVID-19. |  |
| SN | SN1 | My family members think I should get the Covid-19 vaccine | Chu & Liu (2021) |
|  | SN2 | My close friends think I should get the Covid-19 vaccine |  |
|  | SN3 | People who are important to me will get vaccinated for Covid-19. |  |
|  | SN4 | People who are important to me think that I should get Covid-19 vaccines |  |
|  | SN5 | People who influence my behaviour think that I should get vaccinated for Covid-19 |  |
| Note: ABR – Access barriers; ATT – Attitude; BARRIERS – Perceived barriers; BENEFITS – Perceived benefits; CA – Cues to action; CBR – Clinical barriers; CI – Continuance intention; PU – Perceived usefulness; SA – Satisfaction; SN – Subjective norms. | | | |
