# Supplementary material for: The continuance intention to vaccinate against COVID-19: An empirical study from Malaysia
Source: PLoS One. 2024 Apr 30;19(4):e0301383. doi: 10.1371/journal.pone.0301383 (PMC11060549; doi:10.1371/journal.pone.0301383)
Supplement: S2 Appendix — (DOCX) [file pone.0301383.s002.docx]

## S2 Appendix. FIMIX-PLS results

**Information criteria**

|  | **Segment Size** | | | | |
| --- | --- | --- | --- | --- | --- |
|  | 1 | 2 | 3 | 4 | 5 |
| AIC3 (modified AIC with Factor 3) | 17108.33 | 14775.15 | 14439.07 | 14103.12 | **14046.46** |
| CAIC (consistent AIC) | 17197.24 | 14958.53 | 14716.92 | **14475.43** | 14513.24 |
| EN (normed entropy statistic) |  | 0.863 | 0.72 | 0.743 | 0.693 |

**Segment sizes**

|  | Segment 1 | Segment 2 | Segment 3 | Segment 4 | Segment 5 |
| --- | --- | --- | --- | --- | --- |
| **Segment size (%)** | 1 |  |  |  |  |
|  | 0.638 | 0.362 |  |  |  |
|  | 0.474 | 0.364 | 0.163 |  |  |
|  | 0.385 | 0.348 | 0.164 | 0.103 |  |
|  | 0.346 | 0.296 | 0.189 | 0.105 | 0.064 |
