# Supplementary material for: The continuance intention to vaccinate against COVID-19: An empirical study from Malaysia
Source: PLoS One. 2024 Apr 30;19(4):e0301383. doi: 10.1371/journal.pone.0301383 (PMC11060549; doi:10.1371/journal.pone.0301383)

## S3 Appendix. Tukey’s test

This appendix presents the results of the Tukey’s test, a post hoc test conducted subsequent to the ANOVA presented in Table 3. This test compares the means for each of the nine variables across the three continuance intention categories by plotting the 95% confidence interval for the mean difference between the respective continuance intention categories. The difference between any two group means is statistically significant if the interval does not contain zero.

1. **Clinical Barriers**


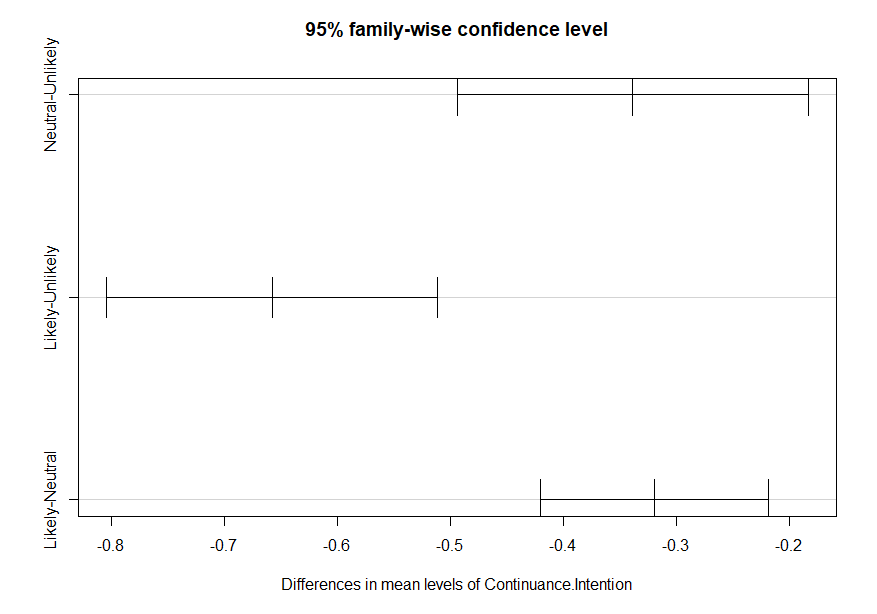


1. **Access Barriers**


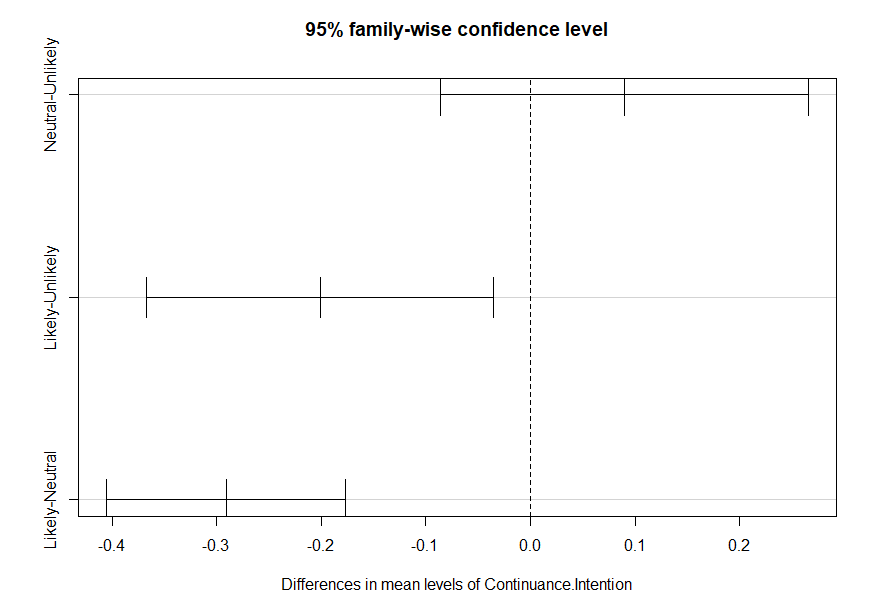


1. **Individual Benefits**


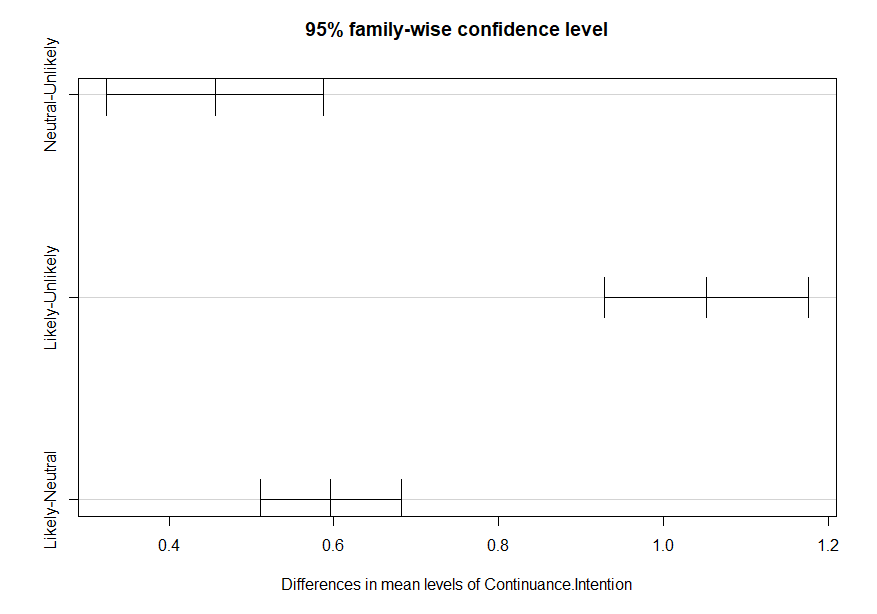


1. **Community Benefits**


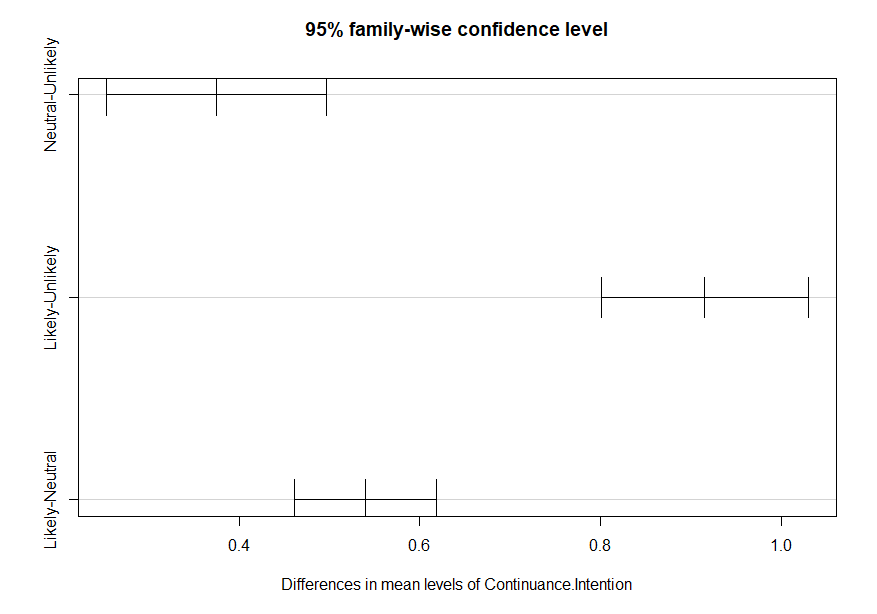


1. **Cues to Action**


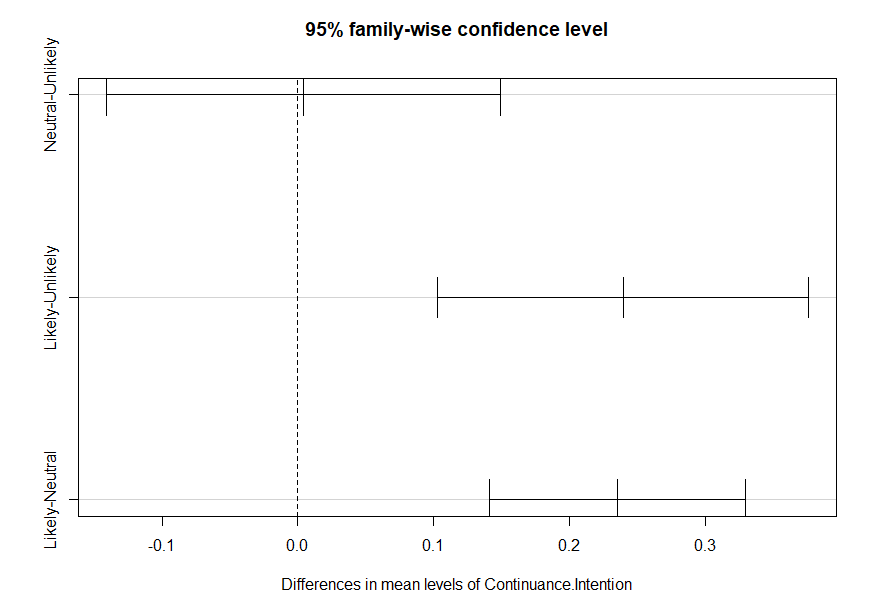


1. **Attitude**


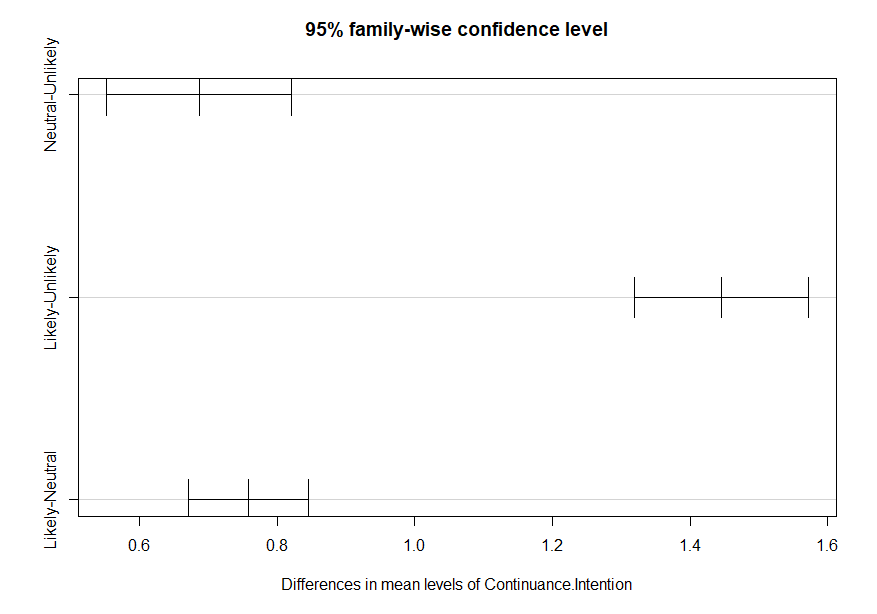


1. **Subjective Norms**


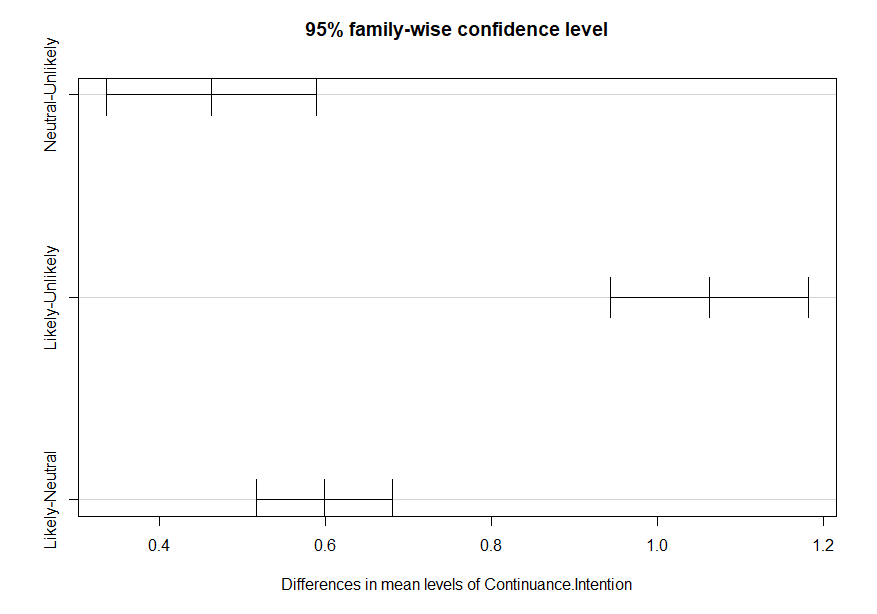


1. **Satisfaction**


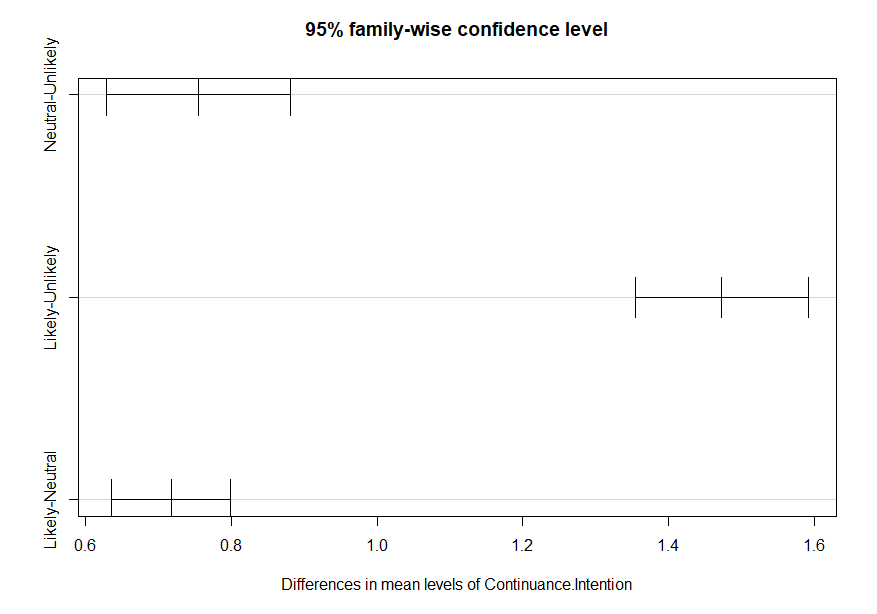


1. **Perceived Usefulness**


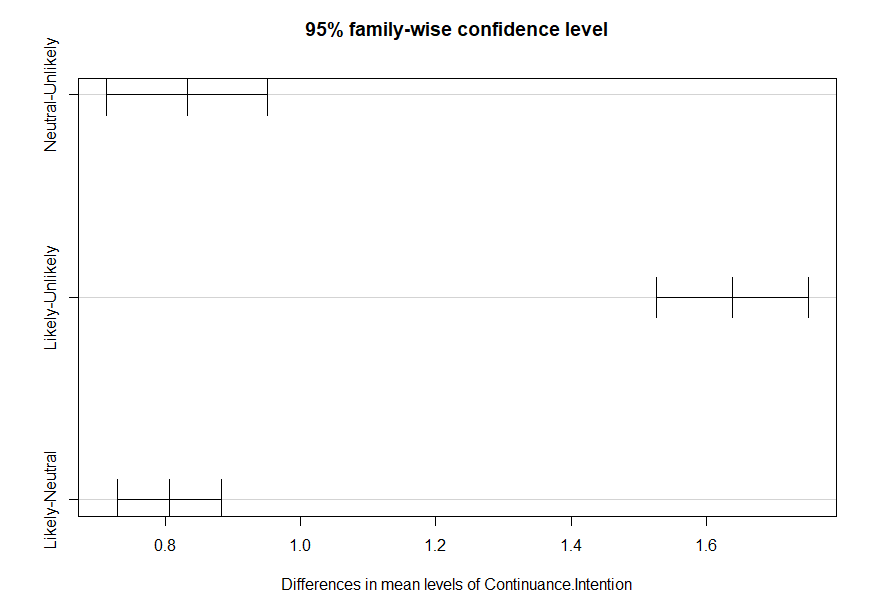

Supplement: S3 Appendix — (DOCX) [file pone.0301383.s003.docx]
