# Supplementary material for: The continuance intention to vaccinate against COVID-19: An empirical study from Malaysia
Source: PLoS One. 2024 Apr 30;19(4):e0301383. doi: 10.1371/journal.pone.0301383 (PMC11060549; doi:10.1371/journal.pone.0301383)
Supplement: S1 Checklist — (DOCX) [file pone.0301383.s004.docx]

STROBE Statement—checklist of items that should be included in reports of observational studies

|  | Item No. | Recommendation | Page  No. | Relevant text from manuscript |
| --- | --- | --- | --- | --- |
| **Title and abstract** | 1 | (*a*) Indicate the study’s design with a commonly used term in the title or the abstract | 1 | The Continuance Intention to Vaccinate Against COVID-19 |
|  |  | (*b*) Provide in the abstract an informative and balanced summary of what was done and what was found | 2 | Vaccination has been one of the most effective preventive strategies to contain the COVID-19 pandemic. However, as the COVID-19 vaccines’ effect wanes off after some time and given their reduced level of protection against mutation strains of the virus, the calls for boosters and second boosters signal the need for continuous vaccination for the foreseeable future. As Malaysia transitions into the endemic phase, the nation’s ability to co-exist with the virus in the endemic phase will hinge on people’s continuance intention to be vaccinated against the virus. Adapting the expectations confirmation model (ECM) to the public health context and in a developing country, this study integrates the ECM with the health belief model (HBM) and the theory of reasoned action (TRA) to examine the inter-relationships of the predictors of people’s continuance intention to vaccinate against COVID-19. |
| Introduction | | | |  |
| Background/rationale | 2 | Explain the scientific background and rationale for the investigation being reported | 4-5 | Nevertheless, there has been mounting evidence that the COVID-19 vaccines’ effect wanes off after some time, whereby the antibody levels decline progressively after the primary round of vaccinations [9-13]. In addition, there is also evidence that the available vaccines cannot offer the same level of protection against mutation strains of the virus [14, 15]. In response to this development, a booster dose has been advocated after the primary vaccination dosage [16, 17]. Furthermore, a second booster for vulnerable persons has recently been recommended by the WHO’s Strategic Advisory Group of Experts on Immunization [18]. Therefore, as the virus continues to evolve and mutate, continuous vaccination is becoming more likely in the near future. This is necessary to avoid a resurgence of infections that may lead to the return of drastic movement control measures that have been shown to cause mental health problems [19-21] and negative economic impacts [22]. |
| Objectives | 3 | State specific objectives, including any prespecified hypotheses | 5 | This study sought to assess and determine the predictors of the Malaysian adult population’s continuance intention to be vaccinated against COVID-19. Unlike an earlier Malaysian study by Wong et al. [6], this study focuses on the continuance intention to receive a COVID-19 vaccine not just limited to the first booster dose but as long as it is needed to keep the pandemic under control. Moreover, the limited studies that have examined the willingness of people to receive the booster dose consider factors such as attitude and satisfaction but do not empirically analyze how these factors relate to their intention to receive the booster dose [28-30]. |
| Methods | | | |  |
| Study design | 4 | Present key elements of study design early in the paper | 5-6 | In contrast, this study analyzes people’s continuance intention to be vaccinated based on the Health Belief Model (HBM), the Theory of Reasoned Action (TRA) and the Expectation Confirmation Model (ECM). Both the HBM and TRA are well-known and empirically supported models of health behavior [31, 32]. On the other hand, the ECM was initially adapted from the consumer behavior literature to theorize a model of continued information systems usage intention [33].  The ECM posits that consumers tend to form a preliminary expectation of a product before purchasing [33]. They will then form perceptions about the performance of the product and evaluate it against their preliminary expectation. Consumers who are satisfied with the product will then develop repurchase intentions towards the product. In the same vein, the ECM can explain continuance intention to receive the COVID-19 vaccine such that individuals’ initial expectations via the primary vaccine doses will allow them to shape their perception towards vaccination. This will result in greater satisfaction and subsequently lead to continuance intention to get vaccinated in the long run. |
| Setting | 5 | Describe the setting, locations, and relevant dates, including periods of recruitment, exposure, follow-up, and data collection | 8 | A nationwide cross-sectional study was conducted in Malaysia from January 17 to January 26, 2022. A locally based international marketing consulting firm was engaged to collect data from Malaysian adults aged 18 years and above using self-administered questionnaires via its online panel. |
| Participants | 6 | (*a*) *Cohort study*—Give the eligibility criteria, and the sources and methods of selection of participants. Describe methods of follow-up  *Case-control study*—Give the eligibility criteria, and the sources and methods of case ascertainment and control selection. Give the rationale for the choice of cases and controls  *Cross-sectional study*—Give the eligibility criteria, and the sources and methods of selection of participants | 8 | A locally based international marketing consulting firm was engaged to collect data from Malaysian adults aged 18 years and above using self-administered questionnaires via its online panel. |
|  |  | (*b*) *Cohort study*—For matched studies, give matching criteria and number of exposed and unexposed  *Case-control study*—For matched studies, give matching criteria and the number of controls per case | N/A |  |
| Variables | 7 | Clearly define all outcomes, exposures, predictors, potential confounders, and effect modifiers. Give diagnostic criteria, if applicable | 9 | Based on the HBM, we incorporated three constructs to measure participants’ perceived barriers and benefits of vaccinating against COVID-19 and cues to action. Perceived benefits is defined as the positive outcomes of getting vaccinated [31]. Perceived barriers, conceptualized as access and clinical barriers, refer to individuals’ assessment of the influences that impede or discourage vaccination [31]. Cues to action refer to the strategies to activate readiness which leads to the execution of the behavior [41]. Items measuring these constructs were obtained from several sources [32, 42, 43].  Drawing upon the TRA, we included items measuring attitude and subjective norms from Chu & Liu [42] and Yang [32]. This study defines attitude as one’s evaluative affect on getting vaccinated for COVID-19 continuously [44]. Subjective norms refer to one’s perception about whether most people who are important to them think they should or should not continuously get the COVID-19 vaccine [44].  In addition to the HBM and TRA, we adapted items from Zhu et al. [7] that measure the ECM constructs given by satisfaction, perceived usefulness, and continuance intention. Satisfaction is defined as the positive experience from completing the primary round of COVID-19 vaccination, while perceived usefulness refers to the utilitarian value of vaccinating against COVID-19 which includes indicators measuring health, well-being, and convenience [7, 33].  We also obtained data about participants' sociodemographic characteristics, such as age, income, education level, employment status, marital status, and whether they had previously been infected with COVID-19. |
| Data sources/ measurement | 8* | For each variable of interest, give sources of data and details of methods of assessment (measurement). Describe comparability of assessment methods if there is more than one group | N/A. This is a survey questionnaire |  |
| Bias | 9 | Describe any efforts to address potential sources of bias | N/A |  |
| Study size | 10 | Explain how the study size was arrived at | 8 | The sample size was determined using the inverse square root method proposed by Kock and Hadaya [40]. Assuming a 5% significance level, the minimum sample size estimated is 1,298. This method of calculating the required minimum sample size is fairly precise for normal and non-normal data [40]. |

Continued on next page

| Quantitative variables | 11 | Explain how quantitative variables were handled in the analyses. If applicable, describe which groupings were chosen and why | N/A |  |
| --- | --- | --- | --- | --- |
| Statistical methods | 12 | (*a*) Describe all statistical methods, including those used to control for confounding | 10 | R version 4.2.1 was used for descriptive analysis where p-values of less than 0.05 were considered statistically significant. Multivariate analysis was performed using the partial least squares structural equation modeling (PLS-SEM) technique given its advantage of the model obtaining high predictive accuracy and concurrently based on causal explanations [45, 46]. SmartPLS 4 was utilized to analyze the interrelationships between the constructs. |
|  |  | (*b*) Describe any methods used to examine subgroups and interactions | N/A |  |
|  |  | (*c*) Explain how missing data were addressed | N/A |  |
|  |  | (*d*) *Cohort study*—If applicable, explain how loss to follow-up was addressed  *Case-control study*—If applicable, explain how matching of cases and controls was addressed  *Cross-sectional study*—If applicable, describe analytical methods taking account of sampling strategy | 10 | Multivariate analysis was performed using the partial least squares structural equation modeling (PLS-SEM) technique given its advantage of the model obtaining high predictive accuracy and concurrently based on causal explanations [45, 46]. |
|  |  | (*e*) Describe any sensitivity analyses | N/A |  |
| Results | | | | |
| Participants | 13* | (a) Report numbers of individuals at each stage of study—eg numbers potentially eligible, examined for eligibility, confirmed eligible, included in the study, completing follow-up, and analysed | N/A |  |
|  |  | (b) Give reasons for non-participation at each stage | N/A |  |
|  |  | (c) Consider use of a flow diagram | N/A |  |
| Descriptive data | 14* | (a) Give characteristics of study participants (eg demographic, clinical, social) and information on exposures and potential confounders | 10-11 | A total of 1,914 respondents took part in the nationwide survey, with the central region having the majority of respondents and the east coast region the smallest (36% vs 9%). Table 1 shows the overall sociodemographic characteristics of the respondents. The ratio of females to males is almost one (0.96), 75% of respondents were aged 50 years or younger, and nearly half (47%) received tertiary education. Over half were Malay respondents (53%) and married (55%), and 14% had been infected with COVID-19 before the survey was conducted. |
|  |  | (b) Indicate number of participants with missing data for each variable of interest | N/A |  |
|  |  | (c) *Cohort study*—Summarise follow-up time (eg, average and total amount) | N/A |  |
| Outcome data | 15* | *Cohort study*—Report numbers of outcome events or summary measures over time | N/A |  |
|  |  | *Case-control study—*Report numbers in each exposure category, or summary measures of exposure | N/A |  |
|  |  | *Cross-sectional study—*Report numbers of outcome events or summary measures | N/A |  |
| Main results | 16 | (*a*) Give unadjusted estimates and, if applicable, confounder-adjusted estimates and their precision (eg, 95% confidence interval). Make clear which confounders were adjusted for and why they were included | N/A |  |
|  |  | (*b*) Report category boundaries when continuous variables were categorized | N/A |  |
|  |  | (*c*) If relevant, consider translating estimates of relative risk into absolute risk for a meaningful time period | N/A |  |

Continued on next page

| Other analyses | 17 | Report other analyses done—eg analyses of subgroups and interactions, and sensitivity analyses | N/A |  |
| --- | --- | --- | --- | --- |
| Discussion | | | | |
| Key results | 18 | Summarise key results with reference to study objectives | 20-23 | - First, the direct path relationship between perceived usefulness and continuance intention to vaccinate appeared to be the strongest. - Second, our results highlight the prominent role of attitude, an integral factor in spearheading behavioral change, that could influence individuals’ continuous intention to vaccinate against COVID-19. - Third, satisfaction has a significant influence on individuals’ continuance vaccination intention. - Fourth, although subjective norms was also significant in predicting continuance intention, its low path coefficient value indicates its diminished role when compared to perceived usefulness, satisfaction and attitude. |
| Limitations | 19 | Discuss limitations of the study, taking into account sources of potential bias or imprecision. Discuss both direction and magnitude of any potential bias | 24 | This study has several limitations. First, data collection was conducted via an online panel skewed towards the urban population. Therefore, the findings may limit generalizability to the overall population. Future research could consider obtaining a representative sample from the rural population for the findings to be generalizable to the overall population. Second, the current study employs a cross-sectional design where an individual’s perception of continuance intention was obtained from a single point in time. Considering this limitation, future studies could seek to undertake a longitudinal study as individual perceptions may change over time due to various factors. A longitudinal study would be able to provide worthwhile insights into individual behavior and external conditions that may affect one’s continuance intention to vaccinate. |
| Interpretation | 20 | Give a cautious overall interpretation of results considering objectives, limitations, multiplicity of analyses, results from similar studies, and other relevant evidence | 24-25 | Although the COVID-19 pandemic will soon enter its fourth year, discussion on vaccine hesitancy is still ongoing, considering the waning effects of vaccines and the emergence of new variants. Continuous vaccination is, therefore, vital to avoid the resurgence of the virus, highlighting the need for vaccine boosters.  This study integrated three theoretical frameworks (i.e., HBM, TRA and ECM) to examine the factors influencing continuous vaccination intention among Malaysians that can provide valuable insights to shape public health strategies to ensure immunity against the virus. Our findings accentuate the importance of satisfaction and perceived usefulness in determining continuous vaccination intention. The findings also revealed the vital role of attitude as an agent of behavioral change among individuals for continuous vaccination. Based on these findings, policymakers and key stakeholders can formulate strategies and interventions to encourage vaccine booster uptake by improving satisfaction and changing individual attitudes by promoting the inherent benefits of vaccines and leveraging the influence of community leaders. |
| Generalisability | 21 | Discuss the generalisability (external validity) of the study results | 24 | First, data collection was conducted via an online panel skewed towards the urban population. Therefore, the findings may limit generalizability to the overall population. Future research could consider obtaining a representative sample from the rural population for the findings to be generalizable to the overall population. |
| Other information | |  | | |
| Funding | 22 | Give the source of funding and the role of the funders for the present study and, if applicable, for the original study on which the present article is based | N/A |  |

*Give information separately for cases and controls in case-control studies and, if applicable, for exposed and unexposed groups in cohort and cross-sectional studies.

**Note:** An Explanation and Elaboration article discusses each checklist item and gives methodological background and published examples of transparent reporting. The STROBE checklist is best used in conjunction with this article (freely available on the Web sites of PLoS Medicine at http://www.plosmedicine.org/, Annals of Internal Medicine at http://www.annals.org/, and Epidemiology at http://www.epidem.com/). Information on the STROBE Initiative is available at www.strobe-statement.org.
